# Supplementary material for: Spontaneous enteric nervous system activity precedes maturation of gastrointestinal motility
Source: bioRxiv. 2023 Aug 6:2023.08.03.551847. Preprint. [Version 1] doi: 10.1101/2023.08.03.551847 (PMC10418201; doi:10.1101/2023.08.03.551847)
Supplement: Supplement 1 [file media-1.pdf]

Fig.1H Clustering events/10 min

**ANOVA summary**	
F	9.88
P value	0.0054
P value summary	**
Significant diff. among means	Yes
R squared	0.6871

**ANOVA table**	SS	DF	MS	F (DFn, DFc	P value
Treatment (between columns)	3.163	2	1.582	F (2, 9) = 9.8	P=0.0054
Residual (within columns)	1.441	9	0.1601		
Total	4.604	11			

**Tukey's multiple compariso**	Mean Diff.	95.00% CI c	Summary	Adjusted P Value
E16.5 vs. E17.5	-2.367	-5.532 to 0.7	ns	0.1749
E16.5 vs. E18.5	-3.6	-7.190 to -0.	*	0.0492
E17.5 vs. E18.5	-1.233	-4.399 to 1.9	ns	0.6107

Fig.1I Minutes clustered/10 min

**ANOVA summary**	
F	4.151
P value	0.0241
P value summary	*
Significant diff. among means	Yes
R squared	0.1917

**ANOVA table**	SS	DF	MS	F (DFn, DFc	P value
Treatment (between columns)	8.091	2	4.046	F (2, 35) = 4	P=0.0241
Residual (within columns)	34.11	35	0.9747		
Total	42.21	37			

**Tukey's multiple compariso**	Mean Diff.	95.00% CI c	Summary	Adjusted P Value
E16.5 vs. E17.5	-0.7274	-1.680 to 0.2	ns	0.1631
E16.5 vs. E18.5	-1.265	-2.346 to -0.	*	0.0187
E17.5 vs. E18.5	-0.538	-1.491 to 0.4	ns	0.3612

Fig.1H High energy events/10 min

**ANOVA summary**	
F	1.409
P value	0.2821
P value summary	ns
Significant diff. among means	No
R squared	0.1901

**ANOVA table**	SS	DF	MS	F (DFn, DFc	P value
Treatment (between columns)	3.6	2	1.8	F (2, 12) = 1	P=0.2821
Residual (within columns)	15.33	12	1.278		

**Tukey's multiple compariso**	Mean Diff.	95.00% CI c	Summary	Adjusted P Value
E16.5 vs. E17.5	-1	-3.132 to 1.1	ns	0.4476
E16.5 vs. E18.5	-1	-2.741 to 0.7	ns	0.3112
E17.5 vs. E18.5	0	-2.132 to 2.1	ns	>0.9999

Fig.2C Clustering events/10 min

**Multiple paired t tests**	Discovery?	P value	ean of Baseli	ean of 1uM T	Difference	E of differenc	t ratio	df	q value
E16.5	No	0.078141	0.4286	0	0.4286	0.202	2.121	6	0.118383
E17.5	No	0.177966	1.5	0	1.5	0.9574	1.567	5	0.179746
E18.5	No	0.051946	2.333	0	2.333	0.9189	2.539	5	0.118383

Fig.2D Minutes clustered/10 min

**Multiple paired t tests**	Discovery?	P value	ean of Baseli	Mean of TTX	Difference	E of differenc	t ratio	df	q value
E16.5	No	0.090842	0.1847	0	0.1847	0.09178	2.012	6	0.13408
E17.5	No	0.132753	0.3298	0	0.3298	0.1838	1.794	5	0.13408
E18.5	No	0.040168	1.194	0	1.194	0.4336	2.753	5	0.121708

Fig.2G High energy events/10 min

**Multiple paired t tests**	Discovery?	P value	ean of Baseli	ean of 1uM T	Difference	E of differenc	t ratio	df	q value
E16.5	No	0.174688	0.3333	0	0.3333	0.2108	1.581	5	0.264652
E17.5	No	0.269703	1.333	0	1.333	0.8819	1.512	2	0.2724
E18.5	No	0.062352	1.333	0	1.333	0.5578	2.39	5	0.188928

Fig.2J Clustering events/10 min

**Multiple unpaired t tests**	Discovery?	P value	lean of Contr	-Cre1;R26R	Difference	E of differenc	t ratio	df	q value
E16.5	Yes	0.003605	3.143	0	3.143	0.8053	3.903	9	0.00182
E17.5	Yes	0.002085	4.333	0	4.333	0.4303	10.07	3	0.00182
E18.5	No	0.242871	1	0	1	0.8103	1.234	11	0.081767

Fig.2K Minutes clustered/10 min

**Multiple unpaired t tests**	Discovery?	P value	lean of Contr	-Cre1;R26R	Difference	E of differenc	t ratio	df	q value
E16.5	Yes	0.001596	1.376	0	1.376	0.309	4.452	9	0.003223
E17.5	No	0.037039	2.715	0	2.715	0.7565	3.589	3	0.03741
E18.5	No	0.225053	0.1862	0	0.1862	0.1449	1.285	11	0.151536
